# Defining the mutation sites in chickpea nodulation mutants PM233 and PM405

**DOI:** 10.1186/s12870-022-03446-7

**Published:** 2022-02-09

**Authors:** Daniel C. Frailey, Qian Zhang, David J. Wood, Thomas M. Davis

**Affiliations:** 1 Department of Agriculture, Nutrition, and Food Systems, University of New Hampshire, Durham, NH 03824 USA; 2 Department of Forest Resources and Environmental Conservation, Virginia Polytechnic Institute and State University, Blacksburg, VA 24061 USA

**Keywords:** Chickpea, Cicer, Variant identification, Nodulation, Mutant

## Abstract

**Background:**

Like most legumes, chickpeas form specialized organs called root nodules. These nodules allow for a symbiotic relationship with *rhizobium* bacteria*.* The rhizobia provide fixed atmospheric nitrogen to the plant in a usable form. It is of both basic and practical interest to understand the host plant genetics of legume root nodulation. Chickpea lines PM233 and PM405, which harbor the mutationally identified nodulation genes *rn*1 and *rn*4, respectively, both display nodulation-deficient phenotypes. Previous investigators identified the *rn*1 mutation with the chickpea homolog of *Medicago truncatula* nodulation gene *NSP2*, but were unable to define the mutant *rn*1 allele. We used Illumina and Nanopore sequencing reads to attempt to identify and characterize candidate mutation sites responsible for the PM233 and PM405 phenotypes.

**Results:**

We aligned Illumina reads to the available desi chickpea reference genome, and did a de novo contig assembly of Nanopore reads. In mutant PM233, the Nanopore contigs allowed us to identify the breakpoints of a ~ 35 kb deleted region containing the *CaNSP2* gene, the *Medicago truncatula* homolog of which is involved in nodulation. In mutant PM405, we performed variant calling in read alignments and identified 10 candidate mutations. Genotyping of a segregating progeny population narrowed that pool down to a single candidate gene which displayed homology to *M. truncatula* nodulation gene *NIN*.

**Conclusions:**

We have characterized the nodulation mutation sites in chickpea mutants PM233 and PM405. In mutant PM233, the *rn*1 mutation was shown to be due to deletion of the entire *CaNSP*2 nodulation gene, while in mutant PM405 the *rn*4 mutation was due to a single base deletion resulting in a frameshift mutation between the predicted RWP-RK and PB1 domains of the NIN nodulation gene. Critical to characterization of the *rn*1 allele was the generation of Nanopore contigs for mutant PM233 and its wild type parent ICC 640, without which the deletional boundaries could not be defined. Our results suggest that efforts of prior investigators were hampered by genomic misassemblies in the *CaNSP2* region of both the desi and kabuli reference genomes.

**Supplementary Information:**

The online version contains supplementary material available at 10.1186/s12870-022-03446-7.

## Background

Legumes are economically important crops and major sources of protein and other nutrients in human diets. Additionally, they are often used as rotation crops because of their symbiotic relationship with nitrogen-fixing *rhizobium* bacteria, which increases soil nitrogen and improves soil fertility. Among pulse crops, which are non-oilseed, legume crops harvested for their dry seeds, chickpea (*Cicer arietinum*) is the third most produced worldwide, behind dry beans and peas [1]. The kabuli chickpea type produces large, cream-colored seeds with thin seed coats, while the desi type produces smaller, dark brown seeds with a thicker seed coat [1]. Both kabuli and desi types are diploid (2n = 2x = 16) [2, 3].

Chickpeas and most other legumes form root nodules, specialized organs that are inhabited by *rhizobium* bacteria. Chickpeas are nodulated by Mesorhizobium species [4]. Nodule formation allows legumes and rhizobia to form a symbiotic relationship. The plant provides carbon and other nutrients to the bacteria, which colonize the nodules, and the rhizobia provide fixed atmospheric nitrogen in a form usable by the plant [5].

The process of nodule formation is called nodulation, and it depends on gene products from both the rhizobia and the plant. Legume roots secrete flavonoids, which induce nodulation (nod) gene expression in the rhizobia. Rhizobial nod genes encode the biosynthetic machinery to synthesize lipo-chitooligosaccharide (LCO) molecules, which are known as Nodulation (Nod) factors. These are detected by the plant and trigger nodule formation. After Nod factor recognition, there are many additional plant genes encoding proteins involved in downstream signaling, nodulation regulation, and metabolic function [6].

Close to a hundred genes involved in nodulation have been discovered in the model legumes, *Medicago truncatula* and *Lotus japonicus* [7] through a combination of forward and reverse genetics studies. Many genes involved in nodulation were originally identified as nodulin genes, i.e., genes with increased expression levels in the nodules compared to other plant tissues [8]. Reverse genetics experiments showed that many of these genes encoded proteins involved in nodulation [9–15]. Of greater relevance to the presence study, forward genetic studies have identified additional genes involved in nodulation [16]. Several *M. truncatula* mutant lines deficient in nodulation were generated by γ-ray irradiation and EMS exposure [17, 18]. The genes responsible for these mutant phenotypes were further characterized in followup studies [19–22].

Comparatively few genetic studies of nodulation have been done in chickpea. Six nodulation-mutant chickpea lines were identified following exposure of desi type parental line P502 (ICC 640) seeds to γ-irradiation, and are presumed to have been radiation-induced [23–26]. These mutants and their respective mutant loci (in parentheses) have been numerically designated as PM233 (*rn*1), PM665 (*rn*2), PM679 (*rn*3), PM405 (*rn*4), PM796 (*rn*5), and PM638 (*Rn7*). In addition, a non-nodulating variant ICC 435 M was reported by other investigators, and assigned the locus name *rn*6 [27–29]. More recently there have been several transcriptomics studies of chickpea root nodules [30–32].

Five of the mutant chickpea lines generated by Davis et al. [23, 24] were the initial subjects of the present study, which ultimately focused on mutants PM233 and PM405. Mutant PM233 fails to form nodules when inoculated with rhizobia and grown in nitrogen-deficient media, and is classified as having a non-nodulation phenotype [23, 33]. In contrast, mutant PM405 can form nodules in the presence of rhizobia, but these nodules are small and white, and the nodulated plants exhibit nitrogen deficiency, on which basis PM405 was classified as having an ineffective nodulation phenotype [25].

Of the seven chickpea nodulation mutants enumerated above, only one has been subjected to any form of molecular analysis. On the basis of linkage analysis [34] and cross-referencing of nodulation gene positions in the available kabuli chickpea [2] and *M. truncatula* [35] reference genomes, Ali et al. [34] identified the chickpea homologue of *M. truncatula Nodulation Signaling Pathway* 2 (*NSP2*) as the likely gene underlying the mutant phenotype seen in PM233. These investigators extracted the wild type chickpea homolog *CaNSP2*, numbered in the available kabuli chickpea reference genome annotation as Ca_13583 [2]; however, they did not recover or define the mutant *rn*1 allele at the DNA sequence level.

We have initiated investigations aimed at illuminating the nodulation gene mutations in the six mutagenically derived chickpea nodulation mutants described above. Resources available for this study included the wild type desi line ICC 640 and its derivative mutant lines, the currently available desi [36] and kabuli [2] chickpea reference genomes, and next generation sequencing data sets that we have generated from the wild type and mutant chickpea lines of interest. The results we report here provide new insights into the molecular basis of defective nodulation in chickpea mutants PM233 and PM405.

## Results

Illumina sequencing yielded data sets (total sequence lengths in parentheses) for PM233 (5.3 Gb), PM405 (12.6 Gb), PM638 (12.8 Gb), PM665 (10.0 Gb), PM796 (8.8 Gb), and wild type ICC 640 (11.0 Gb). Based on the cytometrically estimated 1C genome size of ~ 867 Mb for the desi reference assembly accession ICC 4958 [36, 37]), our Illumina data sets provided genome coverages in the range of approximately 6x (for PM233) to 14x (for PM638).

Nanopore sequencing provided an additional 19.2 Gb (22x coverage) of PM233 sequence and 24.6 Gb (28x coverage) of ICC 640 sequence, as exemplified by the ICC 640 length distribution shown in (Additional file 1: Fig. S1). De novo assembly of the ICC 640 Nanopore reads generated 4860 contigs totaling 584,434,807 bp, with a median length of 35,009 bp and an N50 length of 481,908 bp. Similarly, de novo assembly of the PM233 Nanopore reads generated 10,566 contigs totaling 438,365,021 bp with a median length of 19,712 bp, and an N50 length 85,636 bp.

Variant discovery using Illumina sequence aligned to the ICC 4958 v3.0 desi reference genome [36] identified numerous unique variants in each mutant. Upon implementation of our filtering pipeline, a set of final candidate genes was identified in each of these five mutants (Table 1).

**Table 1:** Outcomes of variant and candidate gene identification pipeline in chickpea mutants

Sample	Filter Criterion						
	Total	CDS	Unique	2 Alleles	Read Depth	Min Allele Frequency	Manual IGV Check
PM233	829,368	12,791	412	411	189	168	15
PM405	1,504,184	10,823	133	130	70	48	10
PM638	1,413,448	11,083	133	128	71	48	11
PM665	2,205,449	10,427	138	135	49	44	13
PM796	1,586,765	10,138	90	87	35	23	6

Legend: The listed variant counts are those remaining after implementation of each respective filtration step. The Total column includes all variants found before filtering. The CDS (coding sequence) column includes remaining variants after filtering only for variants found within coding sequence. The Unique column are all variants found only within a single chickpea line. The 2 Alleles column are variants with 2 alelles. The Read Depth column are variants with a read depth of at least 2. The Min Allele Frequency are variants where the alternate allele (the one not matching to the reference sequence) had a frequency of at least 0.9. The Manual IGV (Integrative Genomics Viewer) Check column included variants confirmed after manual confirmation using IGV (i.e. variants which were present in other chickpea lines but not called as variants were excluded)

For our initial report based upon ongoing analysis of these data, we have chosen to focus on two mutants: PM233 because it had been the subject of a prior study resulting in identification of the *CaNSP2* candidate gene on chickpea chromosome Ca5 [34]; and PM405 because its analysis immediately yielded a promising candidate gene, as detailed below. Findings with respect to the remaining nodulation mutants will be reported as those studies reach fruition.

### PM233

**Fig. 1:**
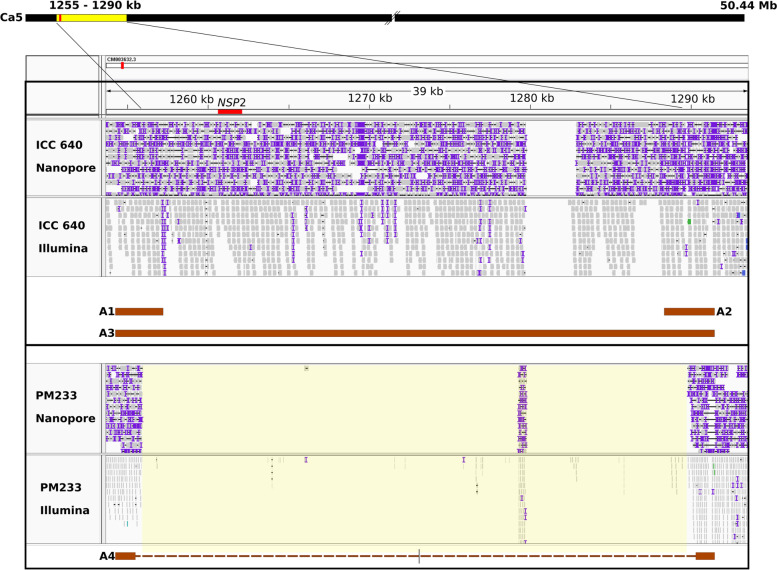
Alignment of ICC 640 (top) and PM233 (bottom) Illumina and Nanopore reads to pseudochromosome Ca5 of the ICC 4958 v3.0 desi reference genome assembly [36], and expected amplicons (brown bars) A1 through A4, as defined in (Additional file 2: Table S1). The location of the *CaNSP2* gene is depicted as a red arrow in the reference track. Its 5′-3′ orientation is left to right. The deletional gap in PM233 is shaded in yellow. The PM233 Nanopore reads that aligned to the left of the deletional gap are not continuous with those that aligned to the right of the gap

Upon their alignment to the ICC 4958 v3.0 desi reference genome, the absence of PM233 Illumina reads aligning to a 35 kb region on chromosome Ca5 that contains the *CaNSP2* candidate gene region (Fig. 1) was immediately apparent, suggesting that this region, ranging from chromosomal coordinates ~ 1255 kb to ~ 1290 kb in the Ca5 reference sequence, was deleted in PM233. There were also several small gaps in the alignment and stretches of N’s in the assembly flanking the putative deletion site, preventing the determination of the precise deletion boundaries using Illumina reads alone (Fig. 1).

Based upon their targeted locations in the ICC 4958 v3.0 desi reference sequence [36], the primer pairs UpF/UpR and DownF/DownR, each consisting of one primer external to, and one primer internal to, the putative deletional gap, were expected to produce amplicons of a predicted size in ICC 640, but no products from PM233 due to deletional loss of internal primer binding sites in the mutant allele. The UpF-UpR primer pair binding to the upstream target worked as expected, producing a PCR product in ICC 640 but not in PM233. However, the DownF-DownR primer pair for the downstream end did not produce an expected product in ICC 640, and the UpF-DownR primer pair did not produce the expected product in PM233 (Additional file 2: Table S1).

**Fig. 2:**
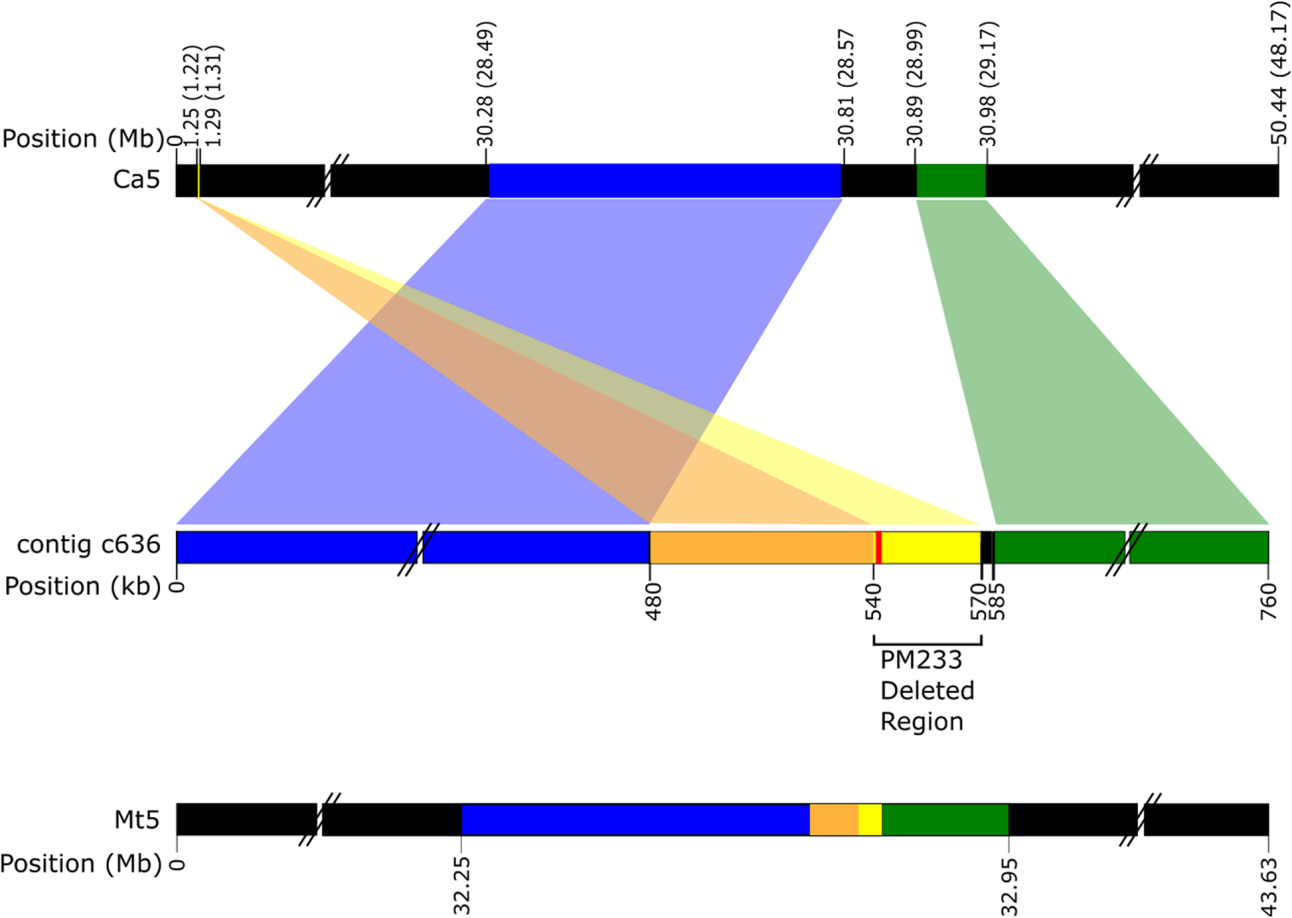
Alignment of genomic region contigs Nanopore ICC 640 contig c636 (middle) to the desi and kabuli (top) and the *M. truncatula* (bottom) reference genomes. The top diagram represents the Ca5 region of interest in both the desi ICC 4958 v3.0 and kabuli CDC Frontier reference genomes, which we found to be collinear with each other in this region. The coordinates along the top bar show the positions in the desi and the kabuli (in parentheses) assemblies, respectively. In the middle diagram, the red bar indicates the *CaNSP2* gene, while the yellow region is the deletional gap within PM233. The orange region is immediately upstream of, and adjacent to, the yellow deleted region and mapped adjacent to the yellow region in both chickpea (top diagram) and the *M. truncatula* version Mt4.0 genome assembly (bottom diagram) assemblies. The sequence block containing the orange and yellow regions and including the deletional region in PM233 resided in between the blue and green regions in contig c636 and in the *M. truncatula* (bottom diagram) assembly, but were positioned elsewhere in the desi and kabuli Ca5 assemblies, as shown (top diagram)

Out of concern that the failure to produce expected amplicons might be attributable to either sequence polymorphism between ICC 640 and ICC 4958, or perhaps an error in the v3.0 reference assembly, we identified informative contigs and reads in our Nanopore-based ICC 640 and PM233 contig assemblies. One of the ICC 640 contigs, number c636 (accession number MZ047314), was ~ 758 kb in length and spanned the entire deletional gap in PM233. When compared to both the ICC 4958 v3.0 desi reference genome [36] and the kabuli reference genome, an internal ~ 90 kb region of contig c636 aligned to the *CaNSP2* candidate gene region on Ca5 of both reference genomes (Fig. 2). However, outside of this region of alignment, the flanking contig segments aligned elsewhere (Fig. 2) on pseudochromosome Ca5, indicative of structural disagreement between our contig c636 and the two reference genomes.

The ~ 90 kb region of alignment between the ICC 640 Nanopore contig c636 and the two chickpea reference genomes encompasses most of the PM233 deletional gap, including the upstream deletion boundary. However, the downstream deletion boundary is outside of this region and is located where the contig and reference assemblies diverge. The PM233 Nanopore reads that aligned to the upstream end of the deletion did not align to the downstream end in the reference assembly (Fig. 1), but did align continuously with the downstream sequence of our contig c636 (not shown).

When our contigs spanning the deletion site c636 and c2 (accession number MZ047315) from ICC 640 (wild type) and PM233 (mutant), respectively, were aligned, they were entirely collinear, except for the contig c2 deletional gap, the position of which is depicted in yellow in contig c636 (Additional file 3: Fig. S2).

### PM405

We did not find any sizable regions of the desi ICC 4958 v3.0 reference assembly that were so poorly covered by PM405 Illumina reads as to suggest any large deletion in PM405. After filtering for read depth, alternate allele frequency, and location within CDS as specified by the ICC 4958 v3.0 genome reference genome [36], we identified 10 sequence variants present only in PM405 and not in ICC 640 or the other nodulation mutant read sets (Table 2). These variants were distributed over four of the eight reference genome pseudochromosomes (Table 2). Of these ten coding sequence variants, eight were substitutions, of which six were synonymous and two were non-synonymous (missense). The remaining two variants were deletions, one within-frame and one causing a frameshift (Table 2). The frameshift mutation consisted of a single base deletion in PM405.

**Table 2:** Coding sequence variants identified in chickpea mutant PM405

Chromosome	GenBank Sequence	Position	Ref	Alt	GeneID	Top tBLASTn Hit	Stop	Frameshift	Syn/NonSyn
Ca2	CM003629.3	37,700,934	G	T	Ca_06416	Heat Shock Protein 90–2	No	No	Syn
**Ca2**	**CM003629.3**	**38,602,731**	**CAA**	**CA**	**Ca_06500**	**Predicted** ***NLP1***	**No**	**Yes**	**NonSyn**
Ca3	CM003630.3	42,275,344	C	T	Ca_09456	Component of oligomeric protein	No	No	NonSyn
Ca6	CM003633.3	19,663,461	A	T	Ca_18764	Glutathione S-transferase-like protein	No	No	Syn
Ca6	CM003633.3	20,028,458	T	G	Ca_05252	Predicted: serine/threonine-protein kinase GRIK2-like	No	No	NonSyn
Ca6	CM003633.3	57,850,387	TAA	–	Ca_20904	Predicted: phosphatidylinositol/phosphatidylcholine transfer protein SFH3	No	No	NonSyn
Ca6	CM003633.3	58,492,000	A	C	Ca_20961	Predicted: 4-coumarate--CoA ligase 1-like	No	No	NonSyn
Ca7	CM003634.3	36,911,781	A	G	Ca_23613	Predicted: ATP-dependent helicase BRM isoform X1	No	No	Syn
Ca7	CM003634.3	6,585,411	G	A	Ca_21616	Uncharacterized	No	No	Syn
Ca7	CM003634.3	7,929,900	G	A	Ca_21755	Uncharacterized	No	No	Syn

Legend. Top tBLASTn hits were taken from the NCBI nucleotide collection database. The position coordinates are those of the ICC 4958 v3.0 genome reference assembly. Ca_06500, the primary candidate gene, is indicated in bold. The Ref (reference) allele is the allele in the ICC 4958 v3.0 genome reference assembly. The Alt (alternate) allele is the allele seen in the PM405 Illumina reads

Each of the ten PM405 variants was in a different gene. When the functional annotations of these ten genes were examined, only one, the Ca_06500 gene at position 38,602,732 on chromosomes Ca2 of the ICC 4958 v3.0 genome assembly had an obvious functional relationship to nodulation. This gene exhibits orthology to the *Medicago* NODULE INCEPTION (NIN) gene, which is known to be essential for nodule formation. The coding sequence mutation in this gene is a single base deletion (CAA - > CA_), and is located at the 2189th nucleotide position of the CDS of a gene annotated as Ca_06500, and produces a frameshift starting at that position (Additional file 4: Fig. S3). This deletion was present in all PM405 reads aligned to the site and none of the reads from ICC 640 or the other mutant lines. The full Ca_06500 gene from start codon to stop codon is 3216 bp and spans from positions 38,600,214 to 38,603,430 on pseudochromosome Ca2 of the reference assembly [36], according to which the CDS covers a total of 2888 bp (Additional file 4: Fig. S3).

The gene annotated as Ca_06500 comprises four exons and three introns (Additional file 4: Fig. S3). When we ran a BLASTn alignment of the Ca_06500 CDS against the *M. truncatula* NCBI database, the top result was the *nodule inception* (*NIN*) gene. The reciprocal BLASTn of the *M. truncatula NIN* gene against the ICC 4958 v3.0 chickpea genome aligned to Ca_06500 as the top result. The chickpea Ca_06500 and *M. truncatula NIN* gene coding regions were aligned in MegAlignPro and shared 72.6% nucleotide sequence identity and 68.5% amino acid identity. The frameshift in PM405 occurs in the fourth exon between predicted RWP-RK and PB1 domains and removes the PB1 domain in the predicted protein encoded by the PM405 allele. On the basis of its potential functional impact, we chose to study this deletion further as a candidate for the *rn*4 mutation in PM405.

In order to assess the association between the Ca_06500 mutation and the ineffective nodulation phenotype in PM405, we phenotyped and genotyped a segregating F2 population from the cross ICC 640 x PM405. Of 24 seeds planted, 19 F2 seeds from this cross germinated to produce F2 plants, which were phenotyped in Leonard Jars using the procedures described. Fourteen of these plants formed normal looking root nodules and five lacked nodules.

In parallel, genotyping of the candidate Ca_06500 mutation site in these 19 F2 plants was accomplished using allele specific primers designed to produce amplicons of differing sizes from the wild type and mutant alleles. A common forward primer (06500F) was used in combination with two different, allele-specific reverse primers (Additional file 5: Table S2, Additional file 6: Fig. S4), 06500Rwt and 06500Rm. The 3′ ends of the allele-specific reverse primers span the single bp deletion, and differ in sequence at that site. The mutation-specific primer 06500Rm also included a 21-nucleotide 5′ tag (Additional file 6: Fig. S4), thus creating an allele-specific mobility shift. This PCR assay (Additional file 5: Table S2) allowed us to genotypically characterize the 19 F2 plants.

PCR using the 06500Rwt reverse primer produced bands in all fourteen nodulating chickpeas and none of the non-nodulating. PCR using the 06500Rm reverse primer produced bands in all five of the non-nodulating plants and nine of the nodulating plants (Additional file 6: Fig. S4). Thus, the normally nodulating F2 plants were of two types: five possessing only the wild type allele, and by inference homozygous; and nine possessing both wild type and mutant alleles as diagnostic of heterozygosity. The five non-nodulating plants possessed only the mutant allele, for which they were presumably homozygous. Within this small data set, the Ca-06500 candidate gene was linked without evident recombination to the nodulation phenotype.

Of the nine other candidate mutations identified in PM405 (Table 2), only one of them was located on the same pseudochromosome as the Ca_06500 gene. Genotyping of this marker established the presence of recombination between this gene and the phenotypically defined locus, excluding this gene from further consideration, as detailed in legend of (Additional file 6: Fig. S4). Based on these analyses, a mutation in the Ca_06500 (*NIN*) gene is indicated as the most likely cause of the phenotype in chickpea mutant PM405.

## Discussion

Our analyses have provided new insights into the identities and phenotypic influences of two chickpea root nodulation genes, PM233 and PM405. The variant calling pipeline identified several (ranging from six to fifteen) mutation sites that fulfilled the filtration criteria in each mutant (Table 1). The respective candidate gene lists are provided here for mutants PM233 (see Additional file 8: Table S4) and PM405 (Table 2), while those for the other three mutants will be presented as components of future studies. Based on the previous report of Ali et al. (2014), our a priori focus in PM233 was the nodulation gene *CaNSP2*, which in both the kabuli [2] and desi [3] reference assemblies is located on pseudochromosome Ca5. However, it was also of interest to apply our variant identification pipeline to PM233 to determine whether mutations in other genes of potential interest might be present, with specific attention to those that might have obvious nodulation functions (if any) located on Ca5. Eight such variant-containing genes were identified on PM233 Ca5 (see Additional file 8: Table S4); three of which were functionally uncharacterized and the other five had no obvious functional connection to nodulation.

### PM233

Here, our focus was to test the hypothesis of Ali et al. (2014) that the *CaNSP2* gene was the site of the *rn*1 mutation conditioning non-nodulation in chickpea mutant PM233, which one of us (Davis) had initially isolated as part of an induced mutagenesis project [23, 24]. Furthermore, our aim was to characterize the *rn*1 mutant allele at the DNA sequence level, where previously only the sequence of the wild type *CaNSP2* (= *Rn*1) allele had been [34]. Using a combination of Illumina and Nanopore sequencing data and constructed contigs from PM233 and wild type ICC 640, we were able to identify and characterize the mutation in PM233 as a ~ 35 kb deletion that completely deletes the *CaNSP2* gene as well as some flanking sequence, but no additional annotated genes. This finding is consistent with the recessive character of the PM233 *rn*1 allele [24, 33]. A possible reason why we were able to define the *rn*1 allele at the DNA sequence level, while Ali et al. [34] were not, is explored in a later section of the Discussion. Confirmation of the identity between *CaNSP2* and *Rn*1 via molecular complementation remains to be accomplished, but was beyond the scope of the present study.

### PM405

Of the ten candidate genes identified by our filtration pipeline in PM405, only one had a functional annotation that suggested a role in root nodulation. The respective mutation site on chromosome Ca2 consisted of a single bp deletion that resulted in a frame shift in a gene identified as Ca_06500 in the ICC 4958 v3.0 assembly. To test the hypothesis that this mutation coincided with the *rn*4 mutation in mutant PM405, we generated a segregating population by crossing PM405 with ICC640 for the purpose of assessing marker-trait linkage association.

Based upon the desi reference sequence [36] corresponding to the mutation site region, we designed allele-specific primers with a WT-Rev primer intended to only amplify the wild type allele, and a Mut-Rev primer intended to only amplify the mutant allele. Based upon this design, a heterozygous individual was expected to produce bands of differing sizes, depending on which of the reverse primers was used. Genotyping of the segregating progeny by allele-specific PCR provided evidence supporting the hypothesis that the observed Ca_06500 mutation is responsible for the nodulation mutant phenotype of PM405. We also genotyped the population for a mutation in a nearby gene, Ca_06416, annotated as Heat Shock Protein 90–4. There were three recombination events between Ca_06500 and Ca_06416 but only one event between Ca_06416 and the *rn4* locus. The *Rn4* allele is dominant so we could only detect recombination events between the candidate gene loci and the *rn4* locus when homozygous recessive (*rn4/rn4*). The recombination event between Ca_06416 and the *Rn4* locus suggests this mutation was not responsible for the non-nodulating phenotype.

Ca_06500 is predicted as an *NLP1* gene through NCBI automatic annotation. However, a BLAST alignment of the CDS against the *M. truncatula* database showed a highest amount of sequence identity with *NIN*. A reciprocal BLAST of *NIN* showed Ca_06500 as the alignment with the highest sequence identity, suggesting Ca_06500 may be an ortholog of *NIN*, not of *NLP1*.

*NIN* expression is induced in *M. truncatula* following Nod factor recognition. The NIN protein contains an RWP-RK-type DNA-binding domain and a Phox and Bem1 (PB1) domain necessary for protein-protein interactions. The predicted protein encoded by Ca_06500 includes the RWP-RK and PB1 domains. The single bp deletion in PM405 occurs between the two domains, and results in loss of PB1 due to a frameshift. Plants with mutations in *NIN* in *M. truncatula* do not form nodules [38].

The mutant segregants in the PM405 segregating population had no nodules, instead of ineffective nodules as previously reported in PM405 [25]. The mutant phenotype is apparently conditional in PM405, and under prior experimental conditions permitted formation of very small, ineffective nodules while under our current experimental conditions the plants were non-nodulating. The nodulation phenotypes of two of the other mutants, PM665 and PM679, were previously shown to also be conditional: both were temperature dependent [24].

### Assembly issues

The ICC 4958 v3.0 desi reference assembly had multiple stretches of N’s and regions of low read coverage in the region containing the PM233 deletion, making it difficult to determine the precise breakpoints using the aligned Illumina reads alone. We designed primers in an attempt to amplify and sequence the two ends of the deletion region, but were unable to amplify the downstream end of the PM233 deletion in ICC 640 using primers designed from the ICC 4958 v3.0 desi assembly. To resolve the deletion breakpoints, we generated our own Nanopore sequencing data from ICC640 and PM233 DNA and performed a de novo assembly of the ICC640 reads. Our assembled contigs agreed with the upstream end of the deleted region of the ICC 4958 v3.0 genome assembly, but diverged from the downstream end. The PM233 Nanopore reads suggest our assembled contig is correct because the same reads align to both the upstream and downstream ends of the deleted region in our assembled wild type contig, while in the ICC 4958 v3.0 genome reference assembly they align only to the upstream end. Our assembled wild type contig also aligns with the *M. truncatula* version Mt4.0 assembly chromosome 5 from ~ 32.25 Mb to ~ 32.95 Mb.

Our contigs assembled from Nanopore reads disagree at the region of the PM233 deletion with both the ICC 4958 v3.0 genome and kabuli reference assemblies. The fact that our c636 contig includes three individual Nanopore reads spanning the entire deletion and also that our contig assembly shows collinearity with the *M. truncatula* version Mt4.0 assembly suggests that the ICC 4958 v3.0 genome and kabuli reference assemblies are misassembled in this region, perhaps due to the abundance of repetitive sequence in the region. This putative misassembly could explain why the PM233 deletion boundaries could not be resolved by previous investigators [34].

## Conclusions

In summary, this study characterized two mutations resulting in nodulation deficient chickpea, confirmed the previously proposed identity of the *Rn*1 gene by defining the nature and boundaries of the *rn*1 mutation in PM233, and identified a candidate gene potentially responsible for the *rn*4 mutation in PM405. Further molecular studies on the identified gene in PM405 could provide more information about the function of this gene. We have also generated Illumina sequencing data from three other nodulation deficient mutant chickpea lines: PM638, PM665, and PM796. Our Nanopore reads and contigs could be used to help identify and characterize mutations in these mutant lines, and could also provide a resource for a next generation desi reference genome assembly.

## Materials & methods

### Plant culture and phenotyping

The original wild type chickpea line ICC 640 was part of a large set of chickpea germplasm obtained by the University of California, Davis from ICRISAT, India in 1981. The nodulation mutant lines PM233, PM405, PM638, PM665, PM679, and PM796 were generated by induced mutagenesis from ICC 640 in 1982 by one of us (T.M. Davis), and have been maintained by T.M. Davis throughout the intervening time period. This information is documented in Davis et al. 1985 [23]. Chickpea plants were grown at the University of New Hampshire Macfarlane Research Greenhouse. Plants grown for leaf tissue and seeds were grown in 8″ diameter plastic pots of ProMix® potting soil on greenhouse benches in natural light. Plants grown for nodulation phenotyping were grown in Leonard jars [39] (see Additional file 9: Fig. S6) in a growth chamber at 25 °C with a photoperiod of 16/8 h and relative humidity of 70%. Leonard jar upper compartments were filled with Whittemore Super Coarse Horticultural Graded Vermiculite, which is N-deficient. The vermiculite was covered with a top layer of perlite to prevent contamination. Assembled Leonard Jars were wrapped in foil and autoclaved prior to use.

*Rhizobium ciceri* TAL 620 (USDA10050) cultures were grown on LMB medium (2 mM Kh_2_PO_4_ + 2 mM Na_2_HPO_4_, 0.8 mM MgSO_4_7H_2_O, 4.5 mM CaCl_2_) and used to inoculate the upper chamber of the Leonard Jar at the time of seed planting. N-deficient nutrient solution (2 mM MgSO_4_, 1 mM K_2_SO_4_, 1 mM K_2_HPO_4_, 2.5 mM CaSO_4_, 0.98 g/L Murashige and Skoog Basal Salts) was supplied to the plants via addition to the lower chamber of each Leonard jar. Plants were uprooted 28 days after planting and phenotyped based on presence or absence of nodules.

### DNA isolation and sequencing

Young leaf tissue was collected from chickpea plants, and DNA was extracted using a modified (see Additional file 10: Supplemental Methods) CTAB extraction method [40]. Isolated genomic DNA was quantified using an Invitrogen Qubit Fluorometer and standardized to 10 ng/μL through dilution with sterile deionized water. For use in Nanopore sequencing, the genomic DNA isolation procedure was performed more gently than for Illumina sequencing, as indicated (see Additional file 3: Supplemental Methods).

Genomic DNA samples from nodulation mutants PM233, PM405, PM638, PM665, and PM796, and from wild type parent ICC 640 were subjected to Illumina sequencing by the Hubbard Center for Genome Studies (HCGS) at the University of New Hampshire (UNH). Illumina libraries for each genotype were prepared using the KAPA HyperPlus Kit, and sequencing was done on a HiSeq 2500 instrument to generate sets of 150 × 150 bp paired end reads. In addition, ICC 640 and PM233 were subjected to Nanopore sequencing on an Oxford Nanopore GridION instrument at the HCGS. Base calling was done by the HCGS using Guppy.

### DNA sequence analysis – Illumina

Illumina reads from each of the five nodulation mutants and wild type ICC 640 were trimmed with Cutadapt [41] and aligned independently to the *Cicer arietinum* desi variety ICC 4958 v3.0 reference genome (NCBI Project Number PRJNA78951) [36, 42] using bwa-mem [43]. Picard Tools [44] was used to clean, sort, and remove duplicate reads from the alignment files.

**Fig. 3:**
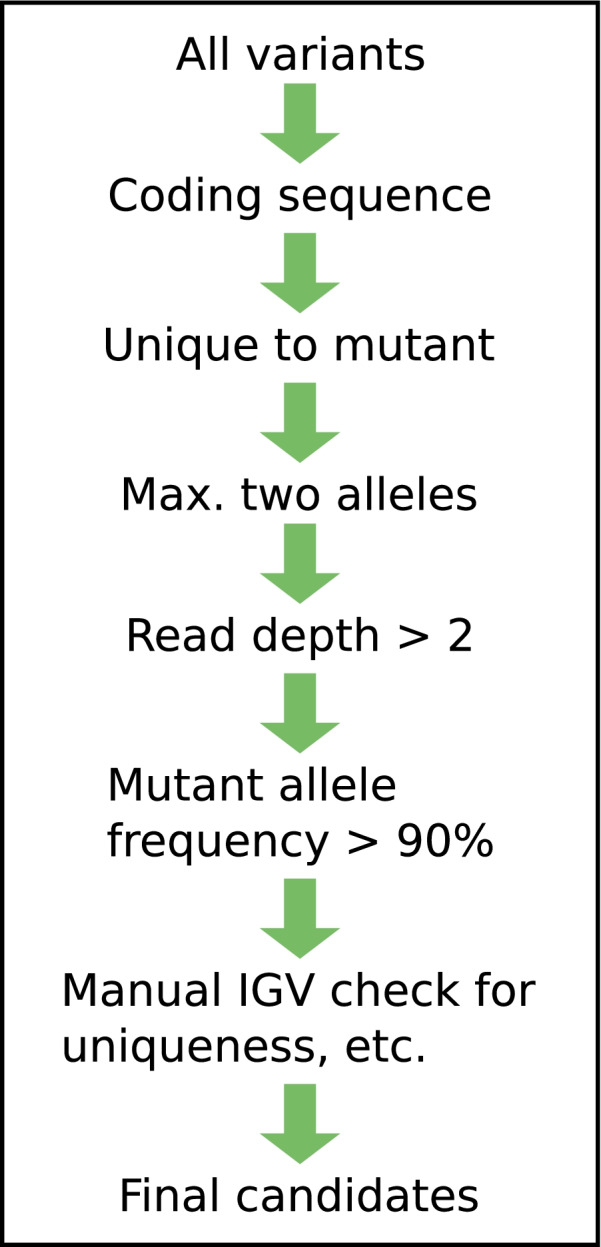
Candidate gene identification pipeline

For the purpose of candidate gene identification, GATK HaplotypeCaller was used to call variants separately in chickpea mutant lines PM233, PM405, PM638, PM665, and PM796, and wild type parent ICC 640. In the variant filtering pipeline (Fig. 3, Additional file 11), variants were retained if they were located within CDS using VCFTools [45], and were unique to the mutant using an in-house Python script (see Additional file 6). Using Microsoft Excel, only variant sites with two alleles, read depth greater than two, and an alternate (non-reference) allele frequency of greater than 0.9 were retained. In a complementary approach, alignments were scanned for occurrence (if any) of large deletions unique to one mutant based on read depth comparisons performed using SAMtools.

The sequential ordering of the filtration steps in the variant identification pipeline is depicted.

The resulting chickpea candidate gene sequences were then aligned to the *M. truncatula* sequence database in NCBI using BLASTn [46]. Chickpea and *M. truncatula* sequences extracted from the respective databases were also aligned with each other using Lasergene MegAlignPro [47].

### DNA sequence analysis and contig assembly - Nanopore

Nanopore sequencing was performed only for mutant PM233 and wild type ICC 640. Adapters were trimmed from Nanopore reads using Porechop [48]. Summary statistics and histograms were generated using NanoPlot [49]. Nanopore reads from ICC 640 and, separately, PM233 were assembled into contigs using the Canu genome assembler [50], followed by polishing with Pilon using the Illumina ICC 640 or PM233 reads, respectively [51]. Nanopore reads from PM233 were then aligned to the assembled ICC 640 contigs, and also to the desi ICC 4958 v3.0 reference assembly [36], using minimap2 [52]. SAM files were sorted and converted to BAM files using SAMtools [53]. Nanopore contigs and specific reads of interest were aligned to the desi ICC 4958 v3.0 reference [36] and kabuli reference [2] assemblies using Mauve [54] and BLASTn [55], and to the *M. truncatula* version Mt4.0 genome [56] downloaded from the *M. truncatula* Genome Database.

#### PCR-based characterization of mutations sites

Primer pairs targeting mutation sites of interest in PM233 and PM405 were initially designed on the basis of ICC 4958 v3.0 reference genome sequence [36] with the aid of Primer3 [57], and later on the basis of our own ICC 640 Nanopore contig sequences as needed. Each reaction consisted of ~ 10 ng template DNA, 2.5 L NEB 10X Standard *Taq* Reaction Buffer, 0.5 μL 10 mM dNTPs, 0.5 μL 10 mM forward primer, 0.5 μL 10 mM reverse primer, and 0.125 μL NEB *Taq* DNA polymerase 10. PCR was performed on an Eppendorf thermocycler. The program was 95 °C for 30 s; 30 cycles of denaturation at 95 °C for 30 s, annealing at 60 °C for 45 s, and elongation at 68 °C for 1 min/kb of expected product size, and a final elongation step at 68 °C for 5 min. Amplicons were visualized on 0.8% agarose (LE Quick Dissolve Agarose) gels.

#### Candidate gene-trait association analysis in PM405

Wild type ICC 640 was crossed as female with PM405 to generate hybrid seeds. A single F1 hybrid plant was allowed to self-pollinate to generate a segregating F2 population, which was subjected to phenotypic screening in Leonard Jars under conditions permitting symbiosis, and to PCR-based genotyping following genomic DNA isolation. Amplicons of mutant and wild type alleles were differentiated by employment of allele-specific primers, or by restriction digests that revealed polymorphisms. Where applicable, restriction digest reactions were set up using 1 μL of NEB *Ava*I restriction enzyme, 10X NEB CutSmart Buffer, and 1 μg of PCR product. Reactions were incubated at 37 °C for 1 h prior to electrophoresis.

## Supplementary Information

**Additional file 1: Fig. S1.** Distribution of ICC 640 Nanopore read lengths. This figure was generated in Nanoplot® [49]. Chickpea accession ICC 640 was sequenced using Nanopore technology. The maximum read length was 175 kb.

**Additional file 2: Table S1.** Primer pairs and their outcomes in the analysis of mutant PM233. The amplicon labels A1-A4 correspond to the expected amplicons depicted in Fig. 1. The primer sequences were designed based on the ICC 4958 v3.0 desi reference genome [36].

**Additional file 3: Fig. S2.** Alignment between assembled genomic region contigs ICC 640 (wild type) contig c636 and PM233 (mutant) contig c2 with coordinates in kb shown. The region colors correspond to those used in Fig. 2. Alignment of these two Nanopore contigs clearly illuminates the PM233 deletion boundaries.

**Additional file 4: Fig. S3.** Chickpea nodulation mutant PM405 Illumina reads aligned to the ICC 4958 v3.0 genome reference assembly as displayed in IGV (Integrated Genomics Viewer). **A)** The entire gene region is shown. The red arrow and box locate the deletion in PM405 reads in the fourth exon. **B**) The same alignment but zoomed in on the region in the red box from **Fig. S3A**.

**Additional file 5: Table S2.** Primer pairs and their outcomes in the analysis of chickpea mutant PM405. Note that the Rm primer has a 21 base 5′ extension relative to the Rwt primer, and also differs at the 3′ base, which is the mutation site. Homozygosity for the wild type (CAA) allele was indicated if the common forward primer produced a band only with the 06500wt reverse primer. Homozygosity for the mutant (CA) allele was indicated if the common forward primer produced a band only with the 06500Rm reverse primer. Heterozygosity was indicated if the predicted products were generated with both primer combinations.

**Additional file 6: Fig. S4.** Genotyping with Ca_06500 allele specific primers. Cropped gel pictures showing PCR results using Ca_06500Rwt primer (left) and Ca_6500Rm reverse primer (right) of WT (ICC 640), PM405, and F2 plants 1, 2, 3, 4, 10, and 11. Plants 2, 3, and 4 had nodules and were phenotyped as WT. Samples 1, 10, and 11 did not have nodules and were phenotyped as mutants. For original, uncropped gel images see Additional file 12: Fig. S6 and Additional file 13: Fig. S7. Ca_06416, annotated as Heat Shock Protein 4, was ~ 900 kb away from Ca_06500 on pseudochromosomes Ca2 in the ICC 4958 v3.0 reference assembly. The mutation was a single base G > T transversion and resulted in loss of an *Ava*I restriction site. For purposes of genotyping, we designed a primer pair targeting this site and ran PCR using DNA from the PM405 segregating population. We subjected the PCR products to restriction enzyme digestion, with the expectation that only the WT allele should be digested, while the PM405 allele would not be digested due to the mutational loss of the *Ava*I site. Recombination was evident between the Ca_06416 candidate gene and both the Ca_06500 gene and the nodulation phenotype. With respect to the two candidate genes, recombination was evident in individuals 3, 7, and 19, each of which was heterozygous for one marker and not the other. With respect to nodulation phenotype, individual 19 was non-nodulating (inferred genotype rn4/rn4) and was heterozygous at the Ca_06416 locus. Five of the nodulating chickpea plants showed complete digestion as would be expected of homozygosity for the wild type allele, and these five plants were also homozygous for the wild type (CAA) allele of the Ca_06500 gene. However, of the ten plants that showed partial digestion, as would be expected for heterozygosity, only seven were also heterozygous at the Ca_06500 locus, while three (plants 3, 7, and 19) were homozygous and therefore counted as recombinants between the Ca_06500 and Ca_06416 loci (Additional file 5: Table S3). Four of the non-nodulating chickpea plants showed no digestion, but one plant (number 19) showed partial digestion, indicative of heterozygosity and therefore of recombination between the Ca_06416 mutation site and the *rn*4 mutation site. On this basis, three instances of recombination between the Ca_06500 and Ca_06416 loci are evident in 19 F2 individuals (Additional file 7: Table S3), representing 2 × 19 = 38 gametes, thus establishing a recombination frequency of 3/38 = 0.079 or about 8%. One instance of recombination is evident between the *rn4* locus and Ca_06416, establishing a recombination frequency of 1/38 = 0.026 or about 3%, while no recombination was detected between the *rn*4 and Ca06500 loci.

**Additional file 7: Table S3.** Results of PCR genotyping analysis of candidate genes Ca_06500 andCa_06416. Three plants (shown in bold) displayed recombination between the two candidate genes, of which one plant (number 19) also displayed recombination between Ca_06416 and the *rn*4 locus.

**Additional file 8: Table S4.** Coding sequence variants identified in chickpea mutant PM233. **Table S4** legend. Top tBLASTn hits were taken from the NCBI nucleotide collection database. The position coordinates are those of the ICC 4958 v3.0 genome reference assembly. The Ref (reference) allele is the allele in the ICC 4958 v3.0 genome reference assembly. The Alt (alternate) allele is the allele seen in the PM233 Illumina reads.

**Additional file 9 **Leonard Jar. **Fig. S6.** The Leonard Jar assembly used in this study. The upper and lower chambers are made from polycarbonate “Magenta ™ GA7 vessels” (Sigma-Aldrich). **A)** Fully assembled Leonard Jar with chickpea seedling. **B)** Fully assembled Leonard Jar without plant or planting substrate. **C)** Disassembled components of Leonard Jar. **1)** Top magenta box with two holes cut in the bottom. **2)** Tubing allowing adding nutrient solution to bottom magenta box. **3)** Bottom magenta box with no holes in it. **4)** Syringe with tip cut off and partially stuffed with cotton. Nutrient solution from the bottom magenta box wicks upward through the syringe into the top magenta box.

**Additional file 10: Supplemental Methods.** CTAB extraction methods used in this study.

**Additional file 11.** Python Script used to filter unique variants.

**Additional file 12 **Cropped Gel 1. **Fig. S7.** Uncropped image of gel from **Fig. S4**.

**Additional file 13 **Cropped Gel 2. **Fig. S8.** Uncropped image of gel from **Fig. S4.**

## Data Availability

Databases used in this study are Pulse Crop Database (https://www.pulsedb.org/) and National Center for Biotechnology Information (https://www.ncbi.nlm.nih.gov/). Public access to both Pulse Crop Database and National Center for Biotechnology Information is open. The datasets generated and/or analyzed during the current study are available in the GenBank repository (https://www.ncbi.nlm.nih.gov/genbank/) under accession numbers MZ047314 and MZ047315. Public access to GenBank is open. The three chickpea lines used in this study have been donated to the USDA Western Regional Plant Introduction Station for maintenance and distribution. These three accessions have been assigned the GRIN Global Accession Numbers (WC Numbers) indicated in parentheses, as follows: mutant PM233B (WC 59438); mutant PM405B (PM 59439); and wild type ICC 640 (WC 59440).

## References

[1] Merga B, Haji J (2019). Economic importance of chickpea: Production, value, and world trade. Cogent Food Agric.

[2] Varshney RK, Song C, Saxena RK, Azam S, Yu S, Sharpe AG, Cannon S, Baek J, Rosen BD, Tar’an B (2013). Draft genome sequence of chickpea (*Cicer arietinum*) provides a resource for trait improvement. Nat Biotechnol..

[3] Jain M, Misra G, Patel RK, Priya P, Jhanwar S, Khan AW, Shah N, Singh VK, Garg R, Jeena G (2013). A draft genome sequence of the pulse crop chickpea (*Cicier arietinum* L.). Plant J..

[4] Greenlon A, Chang PL, Damtew ZH, Muleta A, Carrasquilla-Garcia N, Kim D, Nguyen HP, Suryawanshi V, Krieg CP, Yadav SK (2019). Global-level population genomics reveals differential effects of geography and phylogeny on horizontal gene transfer in soil bacteria. Proc Natl Acad Sci USA..

[5] Udvardi MK, Day DA (1997). Metabolite transport across symbiotic membranes of legume nodules. Annu Rev Plant Physiol Plant Mol Biol..

[6] Suzaki T, Yoro E, Kawaguchi M (2015). Leguminous plants: inventors of root nodules to accommodate symbiotic bacteria. Int Rev Cell Mol Biol..

[7] Roy S, Liu W, Nandety RS, Crook A, Mysore KS, Pislariu CI, Frugoli J, Dickstein R, Udvardi MK (2020). Celebrating 20 years of genetic discoveries in legume noduation and symbiotic nitrogen fixation. Plant Cell..

[8] van Kammen A (1984). Suggested nomenclature for plant genes involved in nodulation and symbiosis. Plant Mol Biol Rep..

[9] Pichon M, Journet EP, Dedieu A, de Billy F, Truchet G, Barker DG (1992). *Rhizobium meliloti* elicits transient expression of the early nodulin gene ENOD12 in the differentiating root epidermis of transgenic alfalfa. Plant Cell..

[10] Asad S, Fang Y, Wycoff KL, Hirsch A (1994). Isolation and characterization of cDNA and genomic clones of MsENOD40; transcripts are detected in meristemic cells of alfalfa. Protoplasma..

[11] Crespi MD, Jurkevitch E, Poriet M, D’Aubenton-Carafa Y, Petrovics G, Kondorosi E, Kondorosi A (1994). ENOD40, a gene expressed during nodule organogenesis, codes for a non-translatable RNA involved in plant growth. EMBO J..

[12] Cook D, Dreyer D, Bonnet D, Howell M, Noney E, VandenBosch K (1995). Transient induction of a peroxidase gene in *Medicago truncatula* precedes infection by *Rhizobium meliloti*. Plant Cell..

[13] Vernoud V, Journet EP, Barker DG (1999). MtENOD20, a nod factor-inducible molecular marker for root cortical cell activation. Mol Plant Microbe Interact..

[14] Endre G, Kereszt A, Kevei Z, Mihacea S, Kaló P, Kiss GB (2002). A receptor kinase gene regulating symbiotic nodule development. Nature..

[15] Combier JP, Frugier F, de Billy F, Boualem A, El-Yahyaoui F, Moreau S, Vernié T, Ott T, Gamas P, Crespi M, Niebel A (2006). MtHAP2–1 is a key transcriptional regulator of a symbiotic nodule development regulated by microRNA169 in *Medicago truncatula*. Genes Dev..

[16] Endre G, Kereszt A, Kevei Z, Mihacea S, Kaló P, Kiss GB. A receptor kinase gene regulating symbiotic nodule development. Nature; 417;962–966. 10.1038/nature00842 12087406

[17] Sagan M, Morandi D, Tarenghi E, Duc G (1995). Selection of nodulation and mycorrhizal mutants in the model plant *Medicago truncatula* (Gaertn.) after γ-ray mutagenesis. Plant Sci..

[18] Catoira R, Galera C, de Billy F, Penmetsa RV, Journet EP, Maillet F, Rosenberg C, Cook D, Gough C, Dénarié J (2000). Four genes of *Medicago truncatula* controlling components of a nod factor transduction pathway. Plant Cell..

[19] Oldroyd GED, Long SR (2003). Identification and characterization of nodulation-signaling pathway 2, a gene of *Medicago truncatula* involved in nod factor signaling. J Plant Physiol..

[20] Smit P, Raedts J, Portyanko V, Debelle F, Gough C, Bisseling T, Geurts R (2008). NSP1 of the GRAS protein family is essential for rhizobial Nod factor-induced transcription. Science..

[21] Kaló P, Gleason C, Edwards A, Marsh J, Mitra RM, Hirsch S, Jakab J, Sims S, Long SR, Rogers J, Kiss GB, Downie JA, Oldroyd GE (2005). Nodulation signaling in legumes requires NSP2, a member of the GRAS family of transcriptional regulators. Science..

[22] Kang Y, Li M, Sinharoy S, Verdier J (2016). A snapshot of functional genetic studies in *Medicago truncatula*. Front Plant Sci..

[23] Davis TM, Foster KW, Phillips DA (1985). Nodulation mutants in chickpea. Crop Sci..

[24] Davis TM, Foster KW, Phillips DA (1986). Inheritance and expression of three genes controlling root nodule formation in chickpea. Crop Sci..

[25] Davis TM (1988). Two genes that confer ineffective nodulation in chickpea (*Cicer arietinum* L.). J Hered..

[26] Paruvangada VG, Davis TM (1999). Brief communication. A dominant, host plant mutation conferring ineffective nodulation in the chickpea-Rhizobium symbiosis. J Hered..

[27] Rupela OP, Sudarshana MR (1986). Identification of a nonnodulation spontaneous mutant in chickpea. Int Checkpea Newsl..

[28] Singh O, Rupela OP, van Rheenen HA (1986). Genetics of nonnodulation in chickpea. Int Chickpea Newsl..

[29] Singh O, Rheenen HAV, Rupela OP (1992). Inheritance of a new non-nodulation gene in chickpea. Crop Sci..

[30] Molina C, Zaman-Allah M, Khan F, Fatnassi N, Horres R, Rotter B, Steinhauer D, Amenc L, Drevon JJ, Winter P, Kahl G (2011). The salt-responsive transcriptome of chickpea roots and nodules via deepSuperSAGE. BMC Plant Biol..

[31] Kant C, Pradhan S, Bhatia S. Dissecting the root nodule transcriptome of chickpea (*Cicer arietinum* L.) PLoS One. 2016;11:e0157908. 10.1371/journal.pone.0157908 PMC4922567 27348121

[32] Kant C, Pandey V, Verma S, Tiwari M, Kumar S, Bhatia S. Transcriptome analysis in chickpea (*Cicer arietinum* L.): Applications in study of gene expression, non-coding RNA prediction, and molecular marker development. In: Marchi FA, PDR C, Mateo EC, editors. Applications of RNA-Seq and Omics Strategies - From Microorganisms to Human Health: IntechOpen; 2017. p. 245–63.

[33] Matthews LJ, Davis TM (1990). Anatomical comparison of wild-type and non-nodulating mutant chickpea (*Cicer arietinum*). Can J Botany..

[34] Ali L, Madrid E, Varshney RK, Azam S, Millan T, Rubio J, Gil J (2014). Mapping and identification of a *Cicer arietinum NSP2* gene involved in nodulation pathway. Theor Appl Genet..

[35] Nayak S, Zhu H, Varghese N, Datta S, Choi HK, Horres R, Jüngling R, Singh J, Kavi Kishor PB, Sivaramakrishnan S (2010). Integration of novel SSR and gene-based SNP marker loci in the chickpea genetic map and establishment of new anchor points with *Medicago truncatula* genome. Theor Appl Genet..

[36] Parween S, Nawaz K, Roy R, Pole AK, Suresh BP, Misra G, Jain M, Yadav G, Parida SK, Tyagi AK (2015). An advanced draft genome assembly of a *desi* type chickpea (*Cicer arietinum* L.). Sci Rep..

[37] Ruperao P, Chan CK, Azam S, Karafiátová M, Hayashi S, Cížková J, Saxena RK, Simková H, Song C, Vrána J (2014). A chromosomal genomics approach to assess and validate the desi and kabuli draft chickpea genome assemblies. Plant Biotechnol J..

[38] Marsh JF, Rakocevic A, Mitra RM, Brocard L, Jongho S, Eschstruth A, Long SR, Schultze M, Ratet P, Oldroyd GED (2007). *Medicago truncatula NIN* is essential for rhizobial-independent nodule organogenesis induced by autoactive calcium/calmodulin-dependent protein kinase. Plant Physiol..

[39] Leonard LT (1943). A simple assembly for use in the testing of cultures of Rhizobia. J Bacteriol..

[40] Torres AM, Wedeen NF, Martin A (1993). Linkage among isozyme, RFLP, and RAPD, markers in *Vicia faba*. Theor Appl Genet..

[41] Martin M (2011). Cutadapt removes adapter sequences from high-throughput sequencing reads. EMBnet J..

[42] Humann JL, Jung S, Cheng C-H, Lee T, Zheng P, Frank M, et al. Cool Season Food Legume Genome Database: A resource for pea, lentil, faba bean and chickpea genetics, genomics and breeding. In: Proceedings of the International Plant and Animal Genome Conference. San Diego: 2019. https://plan.core-apps.com/pag_2019/abstract/2536466f-d222-4b37-ae71-c34238ab3e4b. Accessed 17 Apr 2019.

[43] Li H. Aligning sequence reads, clone sequences and assembly contigs with BWA-MEM. arXiv. 2013; 1303.3997.

[44] Broad Institute. Picard. https://broadinstitute.github.io/picard/. Accessed 5 Feb 2018.

[45] Danecek P, Auton A, Abecasis G, Albers CA, Banks E, DePristo MA, Handsaker R, Lunter G, Marth G, Sherry ST (2011). The variant call format and VCFtools. Bioinformatics..

[46] Altschul SF, Gish W, Miller W, Myers EW, Lipman DJ (1990). Basic local alignment search tool. J Mol Biol..

[47] MegAlign Pro. Version 15.2.0. DNASTAR. Madison, WI. https://www.dnastar.com/. Accessed 8 May 2018.

[48] Wick RR. Porechop. https://github.com/rrwick/Porechop/. Accessed 2 Sept 2019.

[49] De Coster W, D’Hert S, Schultz D, Cruts M, Van Broeckhoven C (2018). Nanopack: visualizing and processing long-read sequencing data. Bioinformatics..

[50] Koren S, Walenz BP, Berlin K, Miller JR, Bergman NH, Phillippy AM (2017). Canu: scalable and accurate long-read assembly via adaptive *k*-mer weighting and repeat separation. Genome Res..

[51] Walker BJ, Abeel T, Shea T, Priest M, Abouelliel A, Sakthikumar S, Cuomo CA, Zeng Q, Wortman J, Young SK, Earl AM (2014). Pilon: an integrated tool for comprehensive microbial variant detection and genome assembly improvement. PLoS ONE..

[52] Li H (2018). Minimap2: pairwise alignment for nucleotide sequences. Bioinformatics..

[53] Li H, Handsaker B, Wysoker A, Fennell T, Ruan J, Homer N, Marth G, Abecasis G, Durbin R (2009). 1000 Genome Project Data Processing Subgroup. The sequence alignment/map format and SAMtools. Bioinformatics..

[54] Darling ACE, Mau B, Blattner FR, Perna NT (2004). Mauve: multiple alignment of conserved genomic sequence with rearrangements. Genome Res..

[55] Camacho C, Coulouris G, Avagyan V, Ma N, Papadopoulos J, Bealer K, Madden TL (2009). BLAST+: architecture and applications. BMC Bioinformatics..

[56] Tang H, Krishnakumar V, Bidwell S, Rosen B, Chan A, Zhou S, Gentzbittel L, Childs KL, Yandell M, Gundlach H (2014). An improved genome release (version Mt4.0) for the model legume *Medicago truncatula*. BMC Genomics..

[57] Untergasser A, Cutcutache I, Koressaar T, Ye J, Faircloth BC, Remm M, Rozen SG (2012). Primer3 – new capabilities and interfaces. Nucleic Acids Res..

