# Supplementary figures and images for: Defining the mutation sites in chickpea nodulation mutants PM233 and PM405
Source: BMC Plant Biol. 2022 Feb 9;22:66. doi: 10.1186/s12870-022-03446-7 (PMC8827291; doi:10.1186/s12870-022-03446-7)

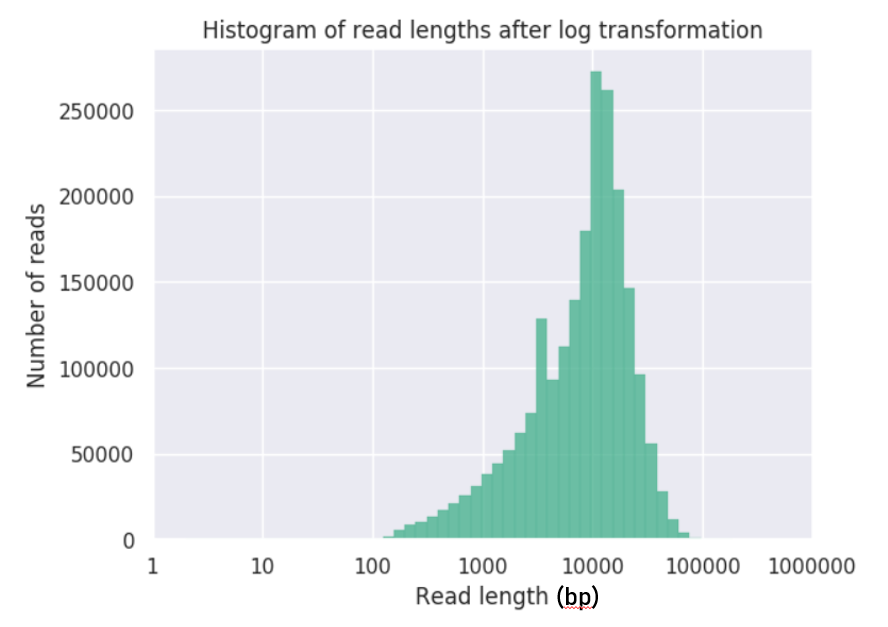

Supplement: Supplementary file 1 — Additional file 1: Fig. S1. Distribution of ICC 640 Nanopore read lengths. This figure was generated in Nanoplot® [49]. Chickpea accession ICC 640 was sequenced using Nanopore technology. The maximum read length was 175 kb. [file 12870_2022_3446_MOESM1_ESM.png]

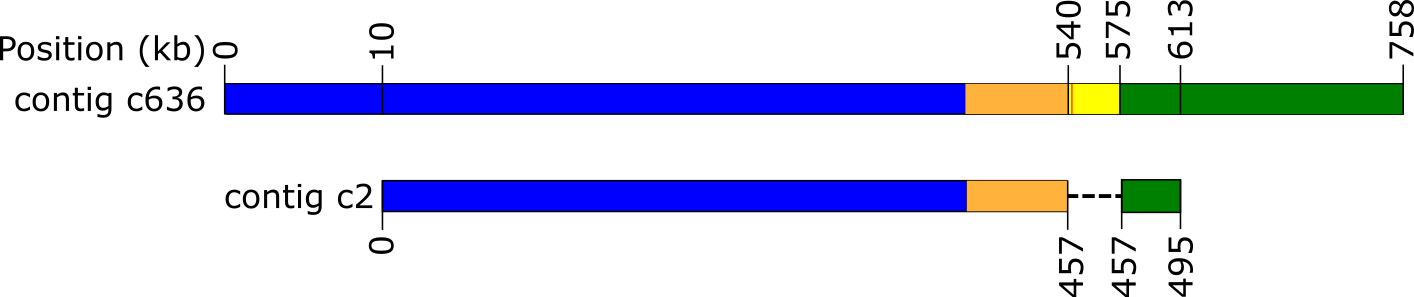

Supplement: Supplementary file 3 — Additional file 3: Fig. S2. Alignment between assembled genomic region contigs ICC 640 (wild type) contig c636 and PM233 (mutant) contig c2 with coordinates in kb shown. The region colors correspond to those used in Fig. 2. Alignment of these two Nanopore contigs clearly illuminates the PM233 deletion boundaries. [file 12870_2022_3446_MOESM3_ESM.png]

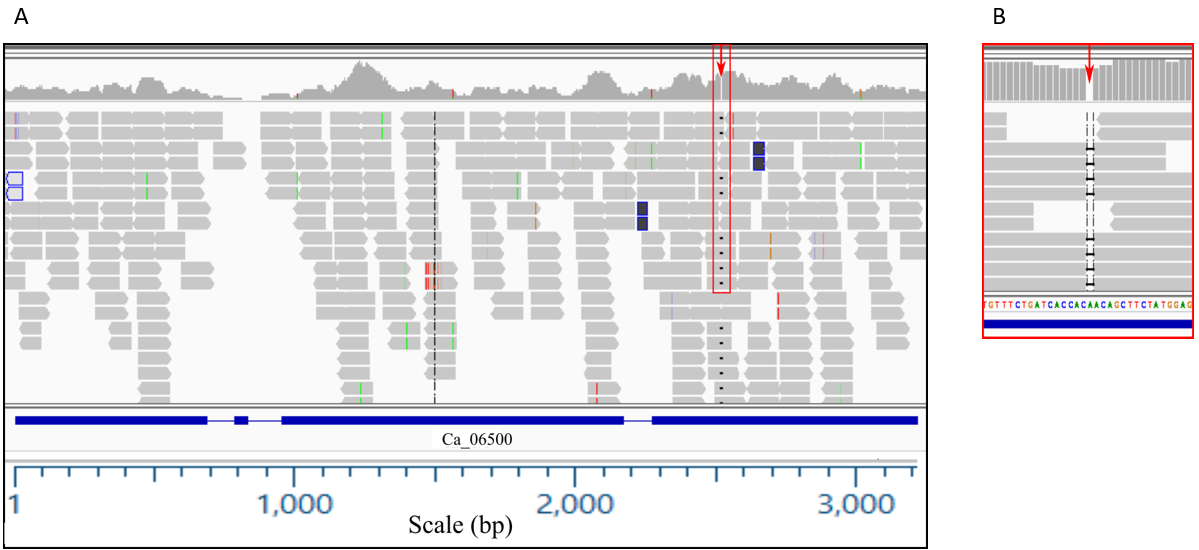

Supplement: Supplementary file 4 — Additional file 4: Fig. S3. Chickpea nodulation mutant PM405 Illumina reads aligned to the ICC 4958 v3.0 genome reference assembly as displayed in IGV (Integrated Genomics Viewer). A) The entire gene region is shown. The red arrow and box locate the deletion in PM405 reads in the fourth exon. B) The same alignment but zoomed in on the region in the red box from Fig. S3A. [file 12870_2022_3446_MOESM4_ESM.png]

## Slide 1
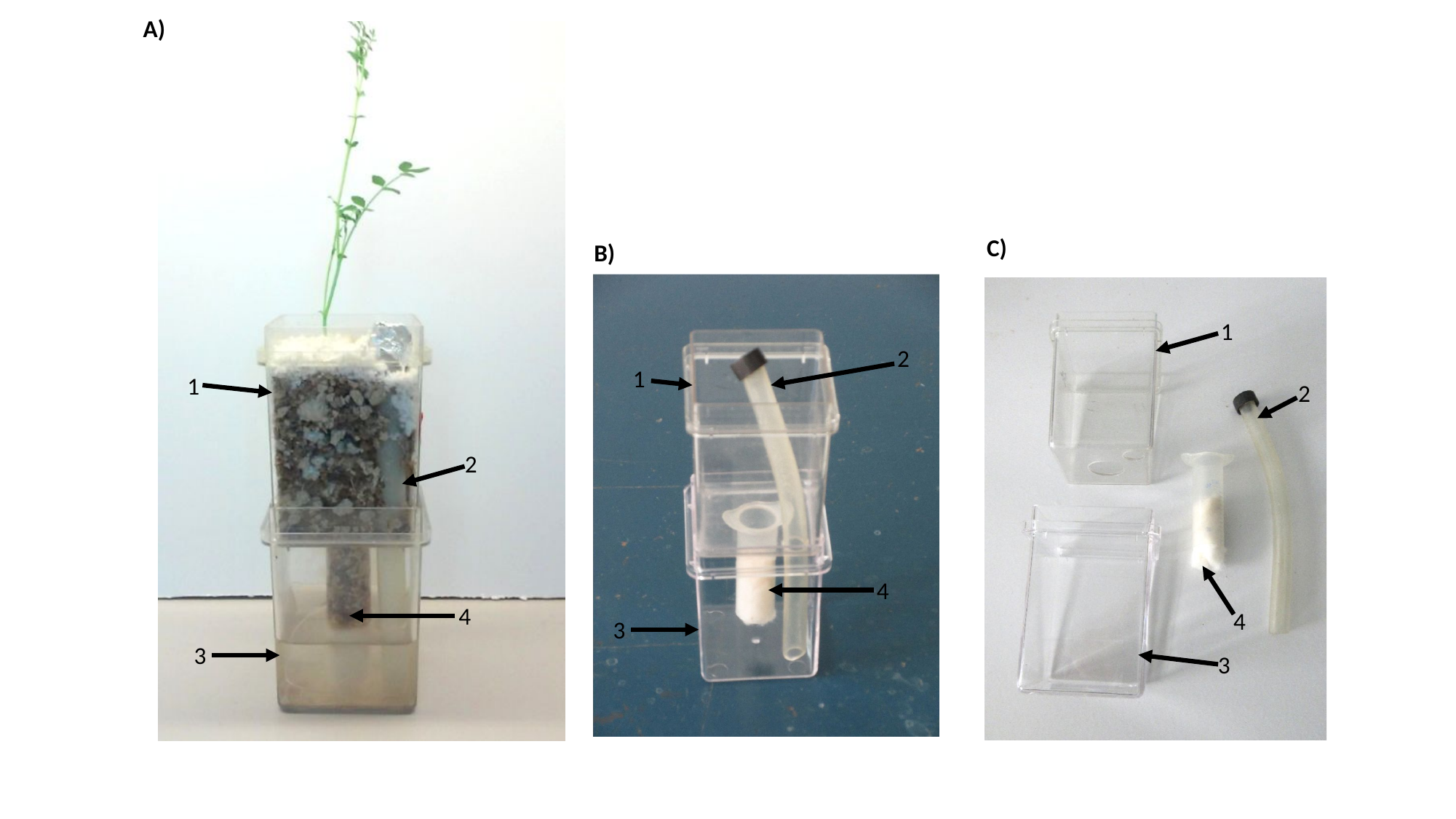

A)
C)
B)
1
2
1
1
2
2
4
4
4
3
3
3

Supplement: Supplementary file 9 — Additional file 9 Leonard Jar. Fig. S6. The Leonard Jar assembly used in this study. The upper and lower chambers are made from polycarbonate “Magenta ™ GA7 vessels” (Sigma-Aldrich). A) Fully assembled Leonard Jar with chickpea seedling. B) Fully assembled Leonard Jar without plant or planting substrate. C) Disassembled components of Leonard Jar. 1) Top magenta box with two holes cut in the bottom. 2) Tubing allowing adding nutrient solution to bottom magenta box. 3) Bottom magenta box with no holes in it. 4) Syringe with tip cut off and partially stuffed with cotton. Nutrient solution from the bottom magenta box wicks upward through the syringe into the top magenta box. [file 12870_2022_3446_MOESM9_ESM.pptx]

## Slide 1
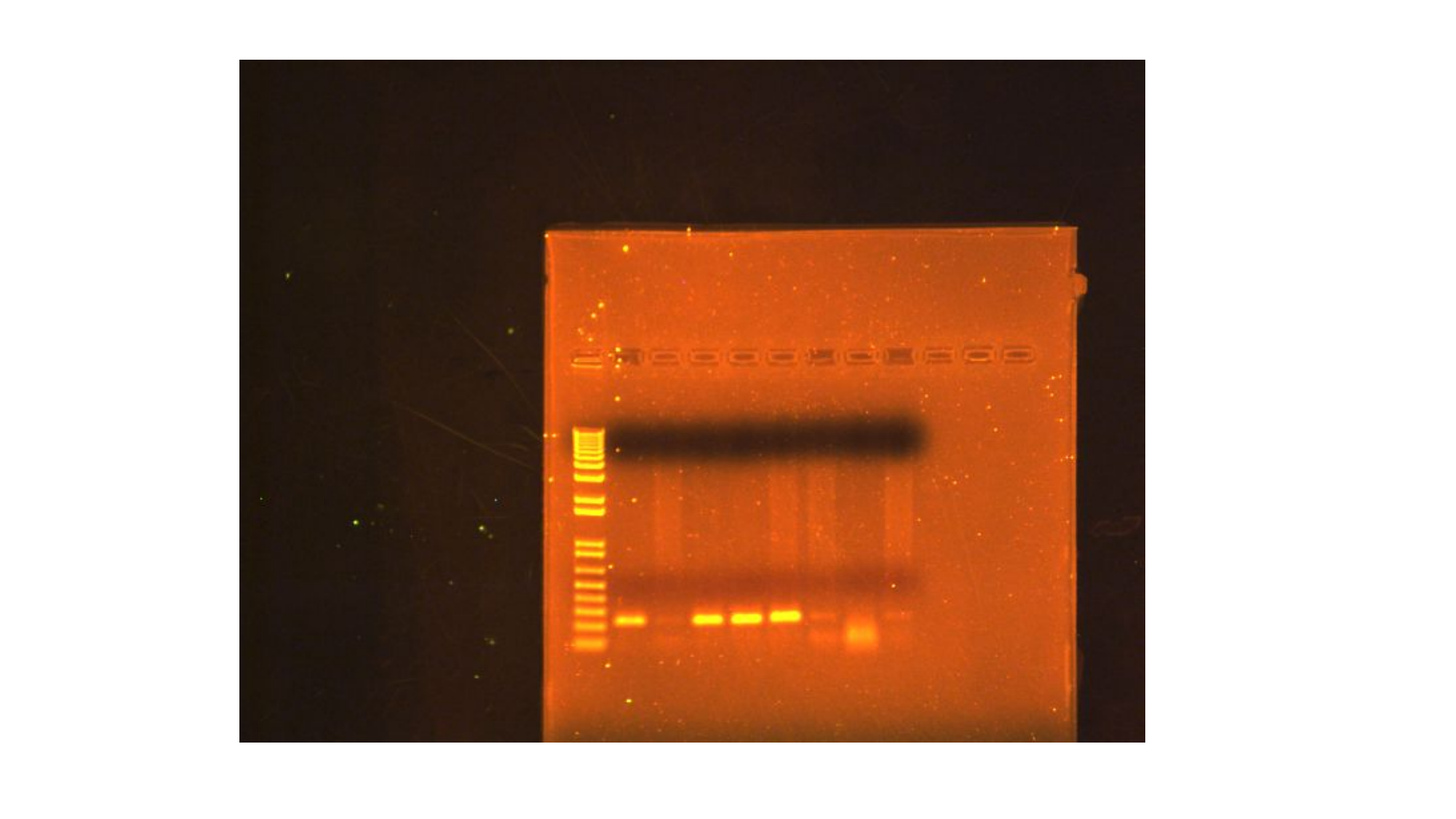

Supplement: Supplementary file 12 — Additional file 12 Cropped Gel 1. Fig. S7. Uncropped image of gel from Fig. S4. [file 12870_2022_3446_MOESM12_ESM.pptx]

## Slide 1
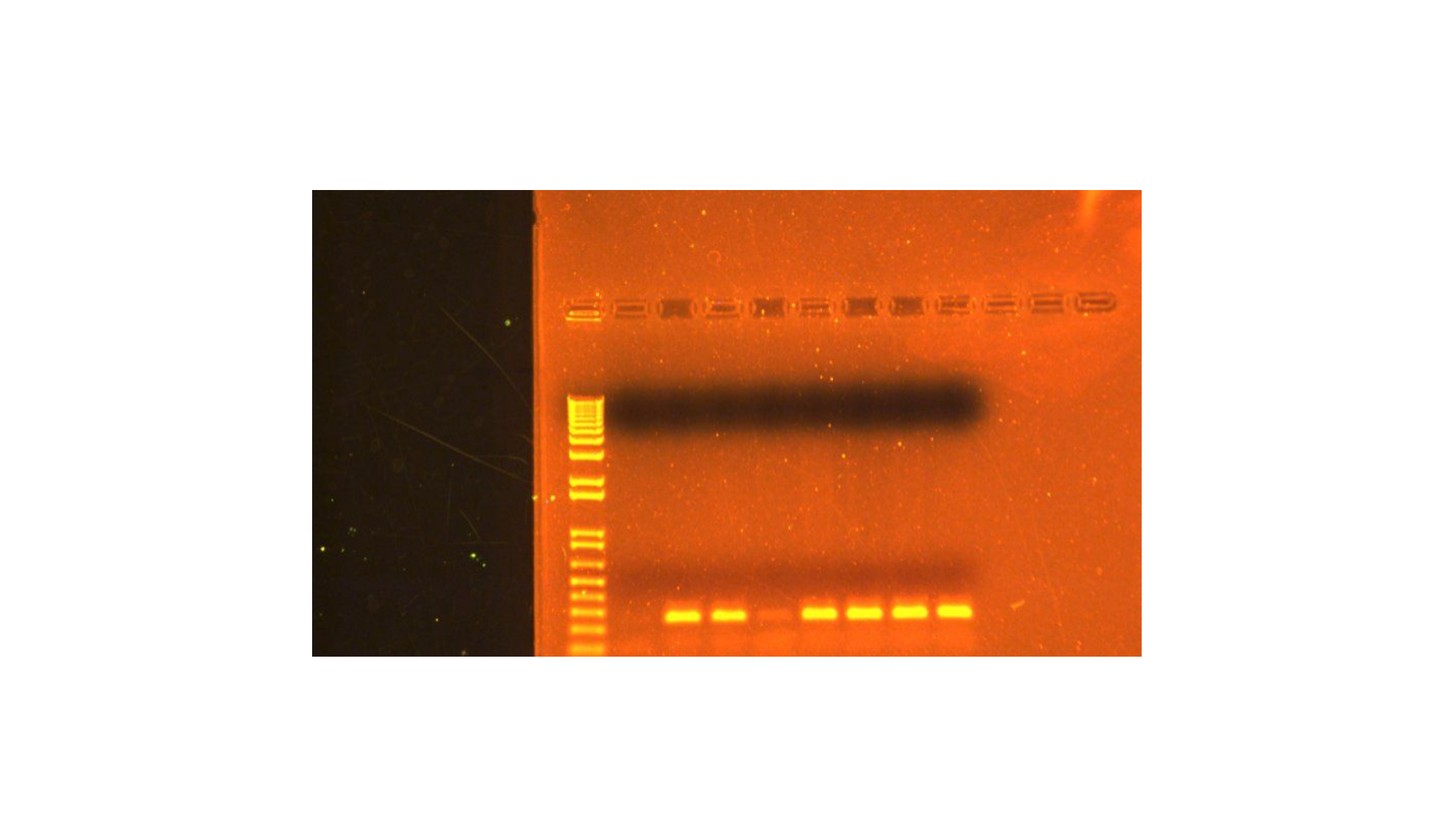

Supplement: Supplementary file 13 — Additional file 13 Cropped Gel 2. Fig. S8. Uncropped image of gel from Fig. S4. [file 12870_2022_3446_MOESM13_ESM.pptx]
