# Supplementary material for: Defining the mutation sites in chickpea nodulation mutants PM233 and PM405
Source: BMC Plant Biol. 2022 Feb 9;22:66. doi: 10.1186/s12870-022-03446-7 (PMC8827291; doi:10.1186/s12870-022-03446-7)
Supplement: Supplementary file 2 — Additional file 2: Table S1. Primer pairs and their outcomes in the analysis of mutant PM233. The amplicon labels A1-A4 correspond to the expected amplicons depicted in Fig. 1. The primer sequences were designed based on the ICC 4958 v3.0 desi reference genome [36]. [file 12870_2022_3446_MOESM2_ESM.docx]

| Primer Pairs | | | ICC 640 | | PM233 | |
| --- | --- | --- | --- | --- | --- | --- |
| Forward | Reverse | | Expected | Observed | Expected | Observed |
| UpF | UpR | | 3.3 kb (A1) | 3.3 kb | None | None |
| DownF | DownR | | 2.5 kb (A2) | None | None | None |
| UpF | DownR | | 35 kb (A3) | None | 300 bp (A4) | None |
|  | | | | | | |
| **Primer sequences** | |  | | | | |
| UpF | | 5'-GCATGTTAAGTTACTCCAACCTTGA | | | | |
| UpR | | 5'-ATCTGGCAAACTTCGCTACC | | | | |
| DownF | | 5'-AAACGTGTTATCGGGTCTCG | | | | |
| DownR | | 5'-GGTACGAACCGGTAGGATCA | | | | |
