# Supplementary material for: Defining the mutation sites in chickpea nodulation mutants PM233 and PM405
Source: BMC Plant Biol. 2022 Feb 9;22:66. doi: 10.1186/s12870-022-03446-7 (PMC8827291; doi:10.1186/s12870-022-03446-7)
Supplement: Supplementary file 5 — Additional file 5: Table S2. Primer pairs and their outcomes in the analysis of chickpea mutant PM405. Note that the Rm primer has a 21 base 5′ extension relative to the Rwt primer, and also differs at the 3′ base, which is the mutation site. Homozygosity for the wild type (CAA) allele was indicated if the common forward primer produced a band only with the 06500wt reverse primer. Homozygosity for the mutant (CA) allele was indicated if the common forward primer produced a band only with the 06500Rm reverse primer. Heterozygosity was indicated if the predicted products were generated with both primer combinations. [file 12870_2022_3446_MOESM5_ESM.docx]

| **Primer Pairs** | | **WT Homozygous**  **CAA/CAA** | | **Heterozygous**  **CAA/CA** | | **Mutant Homozygous**  **CA/CA** | |
| --- | --- | --- | --- | --- | --- | --- | --- |
| Forward | Reverse | Expected | Observed | Expected | Observed | Expected | Observed |
| 06500F | 06500Rwt | 240 bp | 240 bp | 240 bp | 240 bp | None | None |
| 06500F | 06500Rm | None | None | 265 bp | 265 bp | 265 bp | 265 bp |
|  | | | | | | | |
| **Primers** | Primer Sequences | | | | | | |
|  | Forward primer | | | | | | |
| 06500F | 5'-AGACAGCATGGAATAACGAG | | | | | | |
|  | Reverse primers | | | | | | |
| 06500Rwt | 5'-CTCCATCTCCATAGAAGCTGTT | | | | | | |
| 06500Rm | 5'-CAAGCAGAAGACGGCATACGACTCCATCTCCATAGAAGCTGTG | | | | | | |
