# Supplementary material for: Defining the mutation sites in chickpea nodulation mutants PM233 and PM405
Source: BMC Plant Biol. 2022 Feb 9;22:66. doi: 10.1186/s12870-022-03446-7 (PMC8827291; doi:10.1186/s12870-022-03446-7)
Supplement: Supplementary file 6 — Additional file 6: Fig. S4. Genotyping with Ca_06500 allele specific primers. Cropped gel pictures showing PCR results using Ca_06500Rwt primer (left) and Ca_6500Rm reverse primer (right) of WT (ICC 640), PM405, and F2 plants 1, 2, 3, 4, 10, and 11. Plants 2, 3, and 4 had nodules and were phenotyped as WT. Samples 1, 10, and 11 did not have nodules and were phenotyped as mutants. For original, uncropped gel images see Additional file 12: Fig. S6 and Additional file 13: Fig. S7. Ca_06416, annotated as Heat Shock Protein 4, was ~ 900 kb away from Ca_06500 on pseudochromosomes Ca2 in the ICC 4958 v3.0 reference assembly. The mutation was a single base G > T transversion and resulted in loss of an AvaI restriction site. For purposes of genotyping, we designed a primer pair targeting this site and ran PCR using DNA from the PM405 segregating population. We subjected the PCR products to restriction enzyme digestion, with the expectation that only the WT allele should be digested, while the PM405 allele would not be digested due to the mutational loss of the AvaI site. Recombination was evident between the Ca_06416 candidate gene and both the Ca_06500 gene and the nodulation phenotype. With respect to the two candidate genes, recombination was evident in individuals 3, 7, and 19, each of which was heterozygous for one marker and not the other. With respect to nodulation phenotype, individual 19 was non-nodulating (inferred genotype rn4/rn4) and was heterozygous at the Ca_06416 locus. Five of the nodulating chickpea plants showed complete digestion as would be expected of homozygosity for the wild type allele, and these five plants were also homozygous for the wild type (CAA) allele of the Ca_06500 gene. However, of the ten plants that showed partial digestion, as would be expected for heterozygosity, only seven were also heterozygous at the Ca_06500 locus, while three (plants 3, 7, and 19) were homozygous and therefore counted as recombinants between the Ca_06500 and Ca_06 [file 12870_2022_3446_MOESM6_ESM.pptx]

## Slide 1
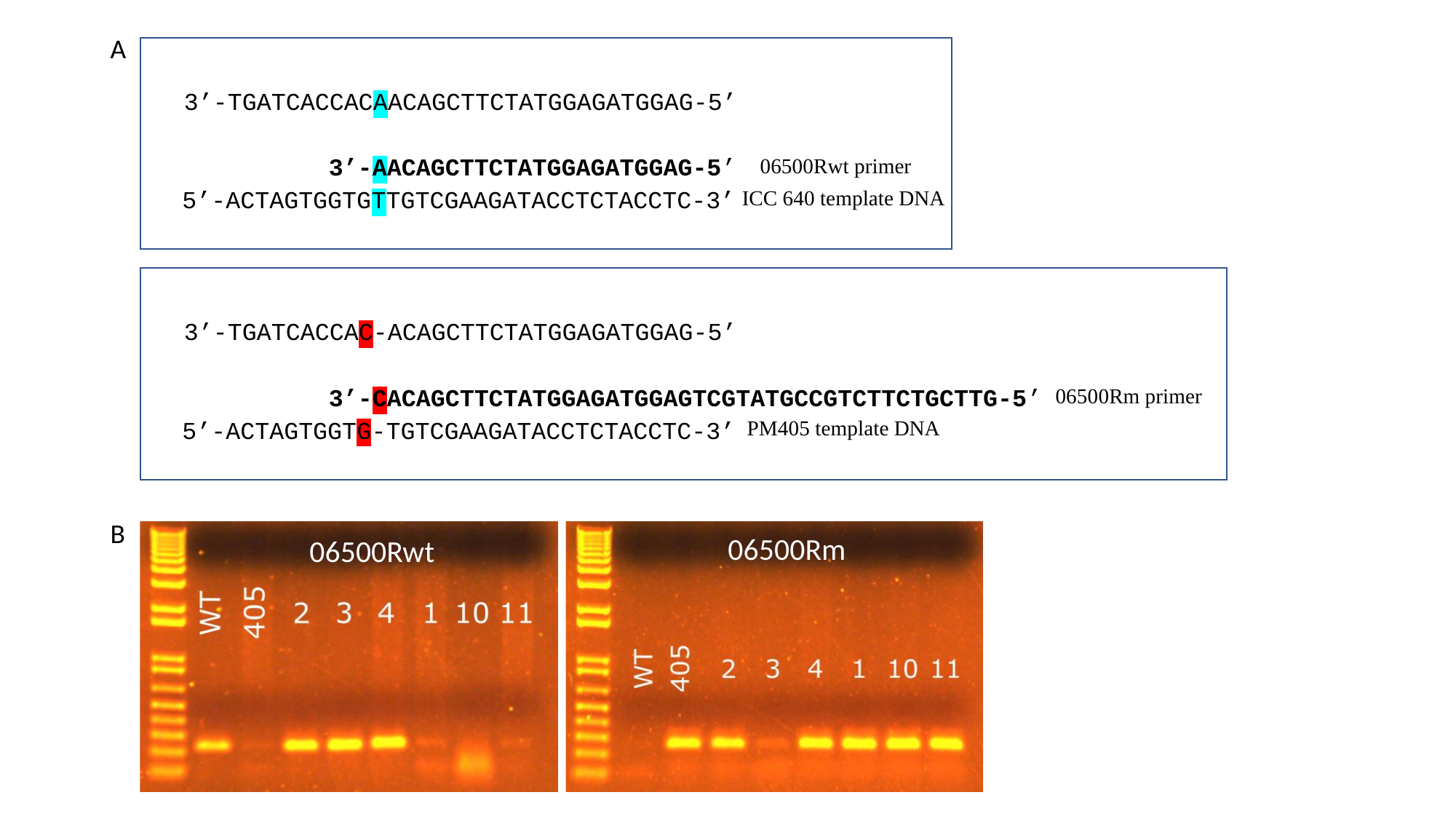

A
3’-TGATCACCACAACAGCTTCTATGGAGATGGAG-5’
3’-AACAGCTTCTATGGAGATGGAG-5’
06500Rwt primer
5’-ACTAGTGGTGTTGTCGAAGATACCTCTACCTC-3’
ICC 640 template DNA
3’-TGATCACCAC-ACAGCTTCTATGGAGATGGAG-5’
3’-CACAGCTTCTATGGAGATGGAGTCGTATGCCGTCTTCTGCTTG-5’
06500Rm primer
5’-ACTAGTGGTG-TGTCGAAGATACCTCTACCTC-3’
PM405 template DNA
B
06500Rm
06500Rwt
