# Supplementary material for: Defining the mutation sites in chickpea nodulation mutants PM233 and PM405
Source: BMC Plant Biol. 2022 Feb 9;22:66. doi: 10.1186/s12870-022-03446-7 (PMC8827291; doi:10.1186/s12870-022-03446-7)
Supplement: Supplementary file 7 — Additional file 7: Table S3. Results of PCR genotyping analysis of candidate genes Ca_06500 andCa_06416. Three plants (shown in bold) displayed recombination between the two candidate genes, of which one plant (number 19) also displayed recombination between Ca_06416 and the rn4 locus. [file 12870_2022_3446_MOESM7_ESM.docx]

| **Sample** | **Phenotype** | **Genotype (Rn4/rn4)** | **Ca_06500 Candidate** | | | | **Ca_06416 Candidate** | | | |
| --- | --- | --- | --- | --- | --- | --- | --- | --- | --- | --- |
|  |  |  | **06500R primer** | | **Inferred genotype** | **Recomb.** | ***Ava*I Digestion** | **Inferred genotype** | **Recomb.** | **Recomb.** |
|  |  |  | **wild type (+)** | **mutant(-)** |  | **Rn4 vs Ca_06500** | **Complete/Partial/No** |  | **Ca_06500 vs Ca_06416** | **Rn4 vs Ca_06416** |
| 1 | Non-Nod | rn4/rn4 | No | Yes | -/- | No | No | -/- | No | No |
| 2 | Nodulating | Rn4/_ | Yes | Yes | +/- | No | Partial | +/- | No | No |
| **3** | **Nodulating** | **Rn4/_** | **Yes** | **No** | **+/+** | **No** | **Partial** | **+/-** | **Yes** | **No** |
| 4 | Nodulating | Rn4/_ | Yes | Yes | +/- | No | Partial | +/- | No | No |
| 5 | Nodulating | Rn4/_ | Yes | No | +/+ | No | Complete | +/+ | No | No |
| 6 | Nodulating | Rn4/_ | Yes | No | +/+ | No | Complete | +/+ | No | No |
| **7** | **Nodulating** | **Rn4/_** | **Yes** | **No** | **+/+** | **No** | **Partial** | **+/-** | **Yes** | **No** |
| 8 | Nodulating | Rn4/_ | Yes | Yes | +/- | No | Partial | +/- | No | No |
| 9 | Nodulating | Rn4/_ | Yes | No | +/+ | No | Complete | +/+ | No | No |
| 10 | Non-Nod | rn4/rn4 | No | Yes | -/- | No | No | -/- | No | No |
| 11 | Non-Nod | rn4/rn4 | No | Yes | -/- | No | No | -/- | No | No |
| 12 | Nodulating | Rn4/_ | Yes | No | +/+ | No | Complete | +/+ | No | No |
| 13 | Nodulating | Rn4/_ | Yes | Yes | +/- | No | Partial | +/- | No | No |
| 14 | Nodulating | Rn4/_ | Yes | Yes | +/- | No | Partial | +/- | No | No |
| 15 | Nodulating | Rn4/_ | Yes | Yes | +/- | No | Partial | +/- | No | No |
| 16 | Nodulating | Rn4/_ | Yes | No | +/+ | No | Complete | +/+ | No | No |
| 17 | Non-Nod | rn4/rn4 | No | Yes | -/- | No | No | -/- | No | No |
| 18 | Nodulating | Rn4/_ | Yes | Yes | +/- | No | Partial | +/- | No | No |
| **19** | **Non-Nod** | **rn4/rn4** | **No** | **Yes** | **-/-** | **No** | **Partial** | **+/-** | **Yes** | **Yes** |
