# Supplementary material for: Defining the mutation sites in chickpea nodulation mutants PM233 and PM405
Source: BMC Plant Biol. 2022 Feb 9;22:66. doi: 10.1186/s12870-022-03446-7 (PMC8827291; doi:10.1186/s12870-022-03446-7)
Supplement: Supplementary file 8 — Additional file 8: Table S4. Coding sequence variants identified in chickpea mutant PM233. Table S4 legend. Top tBLASTn hits were taken from the NCBI nucleotide collection database. The position coordinates are those of the ICC 4958 v3.0 genome reference assembly. The Ref (reference) allele is the allele in the ICC 4958 v3.0 genome reference assembly. The Alt (alternate) allele is the allele seen in the PM233 Illumina reads. [file 12870_2022_3446_MOESM8_ESM.docx]

| **Chromosome** | **GenBank** | **Position** | **Ref** | **Alt** | **GeneID** | **Top tBLASTn Hit** |
| --- | --- | --- | --- | --- | --- | --- |
| Ca5 | CM003632.3 | 1247950 | C | A | Ca_13581 | Uncharacterized |
| Ca5 | CM003632.3 | 25750952 | C | T | Ca_14581 | RNA-binding protein pno1 |
| Ca5 | CM003632.3 | 34614125 | C | T | Ca_15563 | Transmembrane protein, putative |
| Ca5 | CM003632.3 | 42428471 | A | T | Ca_16278 | Endoglucanase |
| Ca5 | CM003632.3 | 1147857 | C | T | Ca_13573 | Mucin-2-like |
| Ca5 | CM003632.3 | 1147866 | A | G | Ca_13573 | Mucin-2-like |
| Ca5 | CM003632.3 | 11772913 | A | G | Ca_14109 | Uncharacterized |
| Ca5 | CM003632.3 | 2950178 | T | G | Ca_13655 | Uncharacterized |
