# Supplementary material for: Defining the mutation sites in chickpea nodulation mutants PM233 and PM405
Source: BMC Plant Biol. 2022 Feb 9;22:66. doi: 10.1186/s12870-022-03446-7 (PMC8827291; doi:10.1186/s12870-022-03446-7)
Supplement: Supplementary file 10 — Additional file 10: Supplemental Methods. CTAB extraction methods used in this study. [file 12870_2022_3446_MOESM10_ESM.docx]

**Supplemental Methods**

DNA was extracted for PCR using a modification of the CTAB extraction method described by Torres et al. [39]. The samples were frozen in liquid nitrogen and ground in a mortar and pestle, then 1 mL of CTAB buffer (2% CTAB, .1 M Tris, 1.4 M NaCl, 20mM EDTA) was added to 0.1g of ground leaf tissue and further ground during thawing until uniform, after which the slurry was poured into an empty 1.5 mL tube. Tubes were incubated in a 60 °C water bath for 1 hr and briefly inverted every 15 min. Finally, tubes were cooled on ice for 10 min, after which 800 µL chloroform:octanol (24:1) was added to each tube and then vortexed. The tubes were centrifuged at 14,000 x g for 5 min and the upper aqueous phase was transferred to a new tube. 1 mL ice cold 95% ethanol was added to each tube, then the tubes were inverted several times and held at -20 °C for 30 min. The tubes were then centrifuged at 14,000 g for 5 min and the supernatant removed. 1 mL ice cold 70% ethanol was added to each tube and inverted several times. Tubes were spun at 14,000 x g for 5 min and the supernatant removed. Tubes were then placed in a speed vacuum for 1 min. 25 µL TE buffer was added to the remaining precipitate and tubes were stored at 4 °C overnight. 25 µL of 1µL RNase/1 mL sterile water solution was added to the tubes and incubated in a 37 °C water bath for 60 1 hr.

DNA was extracted for Nanopore sequencing using the above protocol with the following modifications. Wide bore pipette tips were used, all vortexing steps were replaced with gently inverting the tubes 6-10 times, and all pipetting was done slowly to avoid shearing of the DNA.
